# The role of circadian rhythm proteins Rev-Erbα and β in the development of neuronal injury after traumatic brain injury

**DOI:** 10.55730/1300-0144.5924

**Published:** 2024-09-19

**Authors:** Mahmud E. PENÇE, Enes DOĞAN, Halil İ. KOÇ, Çiğdem BAYRAKTAROĞLU, Serdar ALTUNAY, Zeynep BALÇIKANLI, Ertuğrul KILIÇ, Mustafa Ç. BEKER

**Affiliations:** 1 Research Institute for Health Sciences and Technologies (SABITA), İstanbul Medipol University, İstanbul, Turkiye; 2 Department of Physiology, Faculty of Medicine, İstanbul Medipol University, İstanbul, Turkiye; 3 Department of Physiology, Faculty of Medicine, İstanbul Medeniyet University, İstanbul, Turkiye

**Keywords:** Circadian rhythm proteins, neuronal survival, Rev-Erb, traumatic brain injury

## Abstract

**Background/aim:**

Circadian rhythm proteins (CRPs) play critical roles in both physiological and pathophysiological conditions, including neurodegenerative disorders. As members of CRPs, the nuclear receptors Rev-Erbα/β regulate circadian rhythm particularly by inhibiting Bmal1 protein and are involved in the neuroinflammation and cell death processes. However, their roles in the development of neuronal injury after traumatic brain injury (TBI) were largely unexplored, and so were investigated in the present study.

**Materials and methods:**

For the induction of TBI, animals were subjected to the cryogenic model of TBI, which is a commonly used animal model and shares essential similarities with cerebral ischemia in terms of pathophysiological cascades. To assess the impact of Rev-Erb proteins on TBI, both Rev-Erbα and Rev-Erbβ proteins were activated or deactivated, and their expression profiles were determined by western blot analyses. Infarct volume and brain swelling were analyzed by cresyl violet staining. Blood–brain barrier (BBB) permeability was analyzed by immunoglobulin G extravasation. Neuronal survival was analyzed by NeuN immunohistochemistry.

**Results:**

Our observations indicate that Rev-Erbβ significantly reduced brain injury after TBI, which was reversed by inhibiting this protein. Not activation but the inhibition of both Rev-Erb proteins increased brain swelling significantly. In addition, both Rev-Erbα and Rev-Erbβ improved BBB permeability and neuronal survival significantly, which were reversed by their inhibitions.

**Conclusion:**

Our results show that Rev-Erbα and particularly Rev-Erbβ play significant roles in the development of neuronal injury after TBI. Our findings suggest that Rev-Erb proteins would be a potential therapeutic target for the treatment of neurodegenerative diseases.

## 1.Introduction

Traumatic brain injury (TBI) is a significant global health problem, primarily affecting young adults and children and emerging as a leading cause of disability and death [1,2]. Worldwide, there are an estimated 939 cases of traumatic brain injury per 100,000 people, with around 5.48 million individuals experiencing severe TBI annually [3].

The pathophysiology of TBI is complex, starting with an immediate primary injury to the brain, followed by a series of secondary injuries that can persist for days [4]. The primary injury occurs at the moment of the initial trauma, causing irreversible tissue loss at the core of the lesion. It can be classified as either penetrating (open-head) or nonpenetrating (closed-head or blunt) based on whether the skull is ruptured. The secondary injury involves a cascade of biochemical, cellular, and molecular events that contribute to further brain damage. Key mechanisms include neuroinflammation, blood–brain barrier (BBB) disruption, excitotoxicity, oxidative stress, cerebral edema, and apoptosis [5]. Understanding these processes is crucial for developing therapeutic interventions to reduce the impact of secondary injuries and improve patient outcomes.

Circadian rhythms, present in all life forms from microorganisms to humans, play a crucial role in aligning a variety of internal physiological processes with the daily cycles of the external environment. These intrinsic oscillations, observed in behaviors, physiological states, and metabolic activities, follow a roughly 24-h cycle, hence the term “circadian,” derived from the Latin “circa diem,” meaning “about a day.” These rhythms primarily regulate the human biological clock and influence various physiological functions [6–8].

At the molecular level, circadian rhythm is regulated by a transcription–translation feedback loop involving several gene-encoded proteins. Core clock proteins are the brain and muscle Arnt-like protein 1 (ARNTL, also known as BMAL1) and circadian locomotor output cycles kaput (CLOCK) protein, which form a heterodimer that activates the transcription of period (PER) and cryptochrome circadian regulator (CRY) genes. The PER and CRY proteins accumulate in the cytoplasm and then translocate back into the nucleus to inhibit the activity of the BMAL1–CLOCK complex [9,10]. Rev-Erb plays a crucial role in the molecular clock mechanism by acting as a transcriptional repressor. It binds to specific DNA sequences called ROR elements (ROREs) to inhibit the expression of BMAL1 and CLOCK, thus contributing to the stability and precision of the circadian rhythm. By repressing these core clock genes, Rev-Erb helps fine-tune the feedback loops that drive the circadian cycles, integrating various physiological and metabolic processes [11,12].

Rev-Erbα (Nr1d1) and Rev-Erbβ (Nr1d2) are the main components of the nuclear receptor (NR) superfamily, playing crucial roles in regulating circadian rhythm, metabolic functions, and immune systems [13,14]. Pharmacological activation of Rev-Erb, for instance, has been shown to inhibit the NF-κB signaling pathway, thereby reducing microglial activation and neuroinflammation [15]. Furthermore, Rev-Erb proteins have been implicated in regulating apoptosis and autophagy, as evidenced by their influence on Cyclin A2, ATG5, and BECN1 gene expression [16]. Notably, deficiency of the NR1D1 gene, encoding Rev-Erbα, exacerbates oxidative stress by increasing reactive oxygen species (ROS) and reducing antioxidative genes, such as catalase (CAT), superoxide dismutase 1 (SOD1), and glutathione peroxidase 4 (GPX4) [17]. Nevertheless, within neurodegenerative diseases, elevated concentrations of Rev-Erb exhibit a protective role by diminishing mitochondrial dysfunction and excitotoxicity, which are characteristic manifestations of secondary damage in TBI [18].

In the literature, there are important studies showing the role of circadian rhythm and Rev-Erbs in neurodegenerative diseases [13,19–24]. However, there is a notable absence of investigations that specifically examine its function within the pathophysiology of TBI. Understanding the molecular mechanisms underlying the effects of Rev-Erb proteins on TBI pathophysiology may pave the way for novel therapeutic strategies, offering hope for improved outcomes in TBI patients. In the present study, we investigated the role of Rev-Erbα and/or Rev-Erbβ proteins either by inhibiting or activating their expression levels after TBI. For this aim, we constructed lentiviral vectors and treated animals with them. Three days after TBI, the expression profiles of Rev-Erbα and Rev-Erbβ were identified by western blot analyses, brain injury and brain swelling were analyzed by cresyl violet staining, BBB permeability was evaluated by IgG extravasation, and neuronal survival was determined by neuronal nuclei (NeuN) staining.

## 2. Materials and methods

### 2.1. Ethics statement

The study was conducted in accordance with the ethics standards of the EU Guidelines on the Care and Use of Laboratory Animals (Directive 2010/63/EU) and in agreement with Türkiye’s Animal Research Ethics Committee local guidelines and legislation. The study was approved by local government authorities (İstanbul Medipol University, Animal Research Ethics Committee; reference number: 03/02/2021-09). All animals were maintained under a constant 12-h light/dark cycle (lights on at 07:00 daily) with ad libitum access to food and water. The investigators remained unbiased throughout all experiments and data analysis stages, as they were blinded to the experimental groups.

### 2.2. Experimental groups

A total of 64 BALB/c mice (8–10 weeks old, weighing 20–25 g) were used. They were divided into 8 different groups to increase or decrease Rev-Erbα/β protein expression levels. The groups were as follows: (i) Lv-GFP (empty lentiviral vector), (ii) Lv-RevErbα (for Rev-Erbα overexpression), (iii) Lv-RevErbβ (for Rev-Erbβ overexpression), (iv) Lv-RevErbα/β (for both Rev-Erbα and Rev-Erbβ overexpression), scRNA (scrambled RNA), (vi) Sh-RevErbα (for Rev-Erbα inhibition), (vii) Sh-RevErbβ (for Rev-Erbβ inhibition), and (viii) Sh-RevErbα/β (for both Rev-Erbα and Rev-Erbβ inhibition). Since viral transfections require approximately 7–10 days to infect the target tissue [25], the mice were subjected to virus injection 10 days before the induction of TBI. Following the 10 days of virus injections, the cold-induced TBI model was performed on all mice. After TBI, the mice were monitored for 72 h. At the end of this period, they were deeply anesthetized and euthanized, and their brains were preserved by freezing with dry ice. Subsequently, the brains were sectioned into 18-μm coronal slices using a cryostat (CM1850; Leica, Germany).

### 2.3. Cloning and preparation of lentivirus

A second-generation lentivirus packaging system was used for efficient vector production. High amounts of viral particles were produced using this system to increase or decrease the levels of Rev-Erbα and Rev-Erbβ. For increasing Rev-Erbα/β protein expression, a vector containing GFP protein expression (Lenti-Ef1-GFP–2A-Puro Vector ABM) was used. To inhibit the expression of Rev-Erbα/β commercially available plasmids from Dharmacon (Nr1d1 mEF1a-TurboRFP shRNA glycerol set and Nr1d2 mEF1a-TurboRFP shRNA glycerol set) were used.

Briefly, the coding sequence of human Rev-Erbα (NCBI Reference Sequence: NM_021724.5) and Rev-Erbβ (NCBI Reference Sequence: NM_ NM_005126.5) were amplified using appropriate primers (Rev-Erbα forward: 5′-AGT CAG TCG ACA TGA CGA CCC TGG ACT CCA AC -3′, Rev-Erbα reverse: 5′-AGT CAG GAT CCT CAC TGG GCG TCC ACC CG -3′, Rev-Erbβ forward: 5′-AGT CAG TCG ACA TGG AGG TGA ATG CAG GAG G -3′, and Rev-Erbβ reverse: 5′-AGT CAT CTA GAT TAA GGG TGA ACT TTA AAG GCC A -3′). The PCR products and the Lenti-Ef1-GFP–2A-Puro vector were treated with restriction enzymes for Nr1D1, SalI (FD0644 Thermo Fisher Scientific), BamHI (FD0054 Thermo Fisher Scientific), Nr1D2, SalI (FD0644 Thermo Fisher Scientific), and XbaI (FD0684 Thermo Fisher Scientific). Next, they were ligated using T4 DNA ligase (EL0014, Thermo Fisher Scientific). After that, the insert was confirmed by sequencing. pMD2.G and psPAX plasmids, which were kindly provided by Dr Didier Trono (Ecole Polytechnique Federale, Lausanne, Switzerland), were used as complementary vectors for packaging of the lentiviral system (12259; 12260, Addgene, UK). ScRNA and inhibition vectors for Rev-Erbα and Rev-Erbβ were purchased from Dharmacon.

Seven million HEK293T cells were seeded in 10-cm petri dishes for virus production. The next day, the cells were transfected with 7 μg of expression plasmids (Lv-GFP, Lv-RevErbα, Lv-RevErbβ, Lv-RevErbα/β, scRNA, Sh-RevErbα, Sh-RevErbβ, or Sh-RevErbα/β), 7 μg of psPAX, and 3.5 μg of pMD2.G using Lipofectamine 3000 (L3000015, Thermo Fisher Scientific). Six hours posttransfection, the culture medium was replaced with fresh DMEM. The medium was harvested 24 and 52 h later, centrifuged at 2000 rpm at 4 °C for 10 min, and filtered through a 0.45-μm filter. After ultracentrifugation at 100,000 × *g* for 2 h, virus pellets were resuspended in Dulbecco’s Phosphate-Buffered Saline (DPBS; P04–3650, Pan Biotech) without calcium and magnesium. The virus aliquots were stored at –80 °C. Virus titration was calculated as described before [25].

### 2.4. Administration of lentiviral vectors

The mice were anesthetized with 2% isoflurane (30% O_2_, remainder N_2_O) and placed in a stereotaxic frame (Stoelting, Illinois, USA). The animals’ skulls were drilled and Lv-GFP, Lv-RevErbα, Lv-RevErbβ, Lv-RevErbα/β, scRNA, Sh-RevErbα, Sh-RevErbβ, or Sh-RevErbα/β vectors (10^8^ lentivirus particles in 2 μL of 0.1 M PBS) were administered into the left hemisphere (ipsilesional) (injection coordinates: bregma 1.0 mm posterior, 1.5 mm lateral, and 0.5 mm deep) via a micro syringe pump (Micro 4; World Precision Instrument, Florida, USA).

### 2.5. Cryogenic model of TBI

To perform brain injury, the animals underwent cold-induced TBI, a commonly used model that mimics essential pathophysiological aspects of trauma [26]. In brief, the mice were anesthetized with 1.5% isoflurane (30% O_2_, remainder N_2_O) (AWN-340150-01, Adeka, Turkey), and their rectal temperature was maintained between 36.5 and 37 °C using a feedback-controlled heating system (K 023152, Harvard, UK). The mice were placed in a stereotaxic device (WPI Instruments, USA) with their heads secured using ear bars and a tooth bar. The skull landmarks were carefully aligned and leveled. After sterilizing the scalp with 70% ethanol, a small incision was made, and the connective tissue was removed. A copper probe cooled with liquid nitrogen (tip diameter 1.5 mm) was applied to the skull (1.0 mm posterior and 1.5 mm lateral to bregma). The copper probe was cooled for 3 min in liquid nitrogen before each application to ensure consistent injury. The probe was then placed on the skull for 60 s, after which it was carefully removed. The wounds were sutured after 1 min, and the mice were returned to their home cages. At 72 h post-TBI, the animals were sacrificed using high-dose anesthesia (4% isoflurane, 30% O_2_, N_2_O). Eighteen micrometer coronal cryosections were used to analyze brain injury, brain swelling, serum IgG extravasation, and neuronal survival.

### 2.6. Cresyl violet staining

Coronal brain sections were obtained at equidistant intervals of 1 mm. Then sections were stained with cresyl violet (C5042; Sigma Aldrich) according to the previously published protocol [20]. Within the sections, the border between injured (I) and noninjured (NI) tissues was delineated using image analysis software (ImageJ; National Institute of Health, Maryland, USA). The infarct area was quantified by subtracting the area of the ipsilesional hemisphere noninfarcted (I_NI_) tissue from that of the contralesional hemisphere (C_H_). Infarct volume was calculated by the (C_N_ – I_NI_) × distance between injured areas. Brain swelling was determined as the volume difference between the ipsilesional hemisphere (I_H_) and the C_H_ and expressed in cubic millimeters (mm^3^).

### 2.7. Blood–brain barrier permeability analysis

With gentle stirring, coronal brain sections were rinsed for 10 min at room temperature in 0.1 M phosphate-buffered saline (PBS) to remove intravascular IgG and were fixed in 4% paraformaldehyde (PFA) [27]. Following the blocking of endogenous peroxidase with methanol/0.3% H_2_O_2_ and immersion in 0.1 M PBS containing 5% bovine serum albumin (BSA) and normal swine serum (1:1000), sections were incubated for 1 h in biotinylated horse anti-mouse IgG (BA-1300–2.2, Vectastain Elite; Vector Labs, California, USA) and stained with an avidin peroxidase kit (PK-7800; Vectastain Elite; Vector Labs) and DAB Substrate Kit (SK-4100; Vector Labs). For reasons of data comparability, all sections were processed in parallel. Sections were scanned, and the integrated density of the ipsilesional and contralesional hemispheres was quantified using the NIH ImageJ software. Afterward, density was compared between the ipsilesional tissue and the contralesional tissue to determine BBB leakage.

### 2.8. Neuronal survival analysis

For the evaluation of neuronal survival, coronal brain sections were fixed with 4% paraformaldehyde (PFA)/0.1 M PBS and incubated at room temperature for 1 h with 0.1 mol/L PBS including 0.3% Triton X-100 (PBS-T)/10% normal goat serum (NGS). Then sections were incubated overnight at 4 °C with Alexa Fluor 488-conjugated monoclonal mouse anti-NeuN (Mab377X; Chemicon). The next day, sections were counterstained with 4′,6-diamidino-2-phenylindole (DAPI) and were analyzed using a confocal microscope (LSM800, Carl Zeiss, Jena, Germany) [28]. NeuN-positive cells were counted in 62,500 μm^2^ cortical areas to determine the neuronal survival rate.

### 2.9. Western blot

Mice cortical tissue from TBI subjects was homogenized in RIPA buffer (R0278-50ML, Sigma Aldrich) supplemented with a 1:100 protease/phosphatase inhibitor cocktail (5782, Cell Signaling) and incubated on ice for 15 min. Lysates were centrifuged (14,000 rpm, 15 min, 4 °C), and the supernatant was collected for protein quantification. Protein concentration was determined via Qubit 3.0 Fluorometer (Q33216, Invitrogen) using the Qubit protein assay kit (Q33211, Invitrogen) with 1:50 sample dilution. For western blotting, 20 μg of protein lysate was mixed with 2X Laemmli buffer (1610737, Bio-Rad) and separated on Mini-PROTEAN TGX Precast gels (4569033, Bio-Rad) using 10X Tris/Glycine/SDS running buffer (1610732, Bio-Rad). Proteins were transferred to PVDF membranes (162-0174, Bio-Rad) using a Trans-Blot Turbo System (1704155 Bio-Rad). Membranes were blocked (5% skim milk in TBS-T, 1 h, RT) and incubated overnight with primary antibodies against Rev-Erbα (13418, Cell Signaling Technology), Rev-Erbβ (398252, Santa Cruz Biotechnology), and β-Actin (4970, Cell Signaling Technology). HRP-conjugated goat anti-rabbit (31460, Thermo Fisher Scientific) or goat anti-mouse (31430, Thermo Fisher Scientific) secondary antibodies were used before visualization with a WesternBright ECL kit (K-12045-D20, Advansta) and Chemidoc MP imaging (1708280, Bio-Rad). Protein expression was quantified relative to β-Actin abundance.

### 2.10. Statistics

Statistical analysis was performed using SPSS (version 15, SPSS Inc., Chicago, USA). Data for the western blot experiments were evaluated using the independent samples t-test to compare means between two groups. Data for infarct volume, brain swelling, BBB leakage, and neuronal survival were evaluated using one-way ANOVA to compare means across multiple groups. One-way ANOVA was chosen because it is suitable for comparing the means of three or more independent groups, and our data met the assumptions of normality and homogeneity of variances, as confirmed by the Shapiro–Wilk and Levene’s tests, respectively. Data are presented as mean ± SD values. Exact p-values and effect sizes (partial eta squared) are provided to enhance transparency and replicability. Throughout the study, p-values <0.05 were considered statistically significant.

## 3. Results

### 3.1. Expression profiles of Rev-Erbα and β after lentiviral vector application

**Figure 1:**
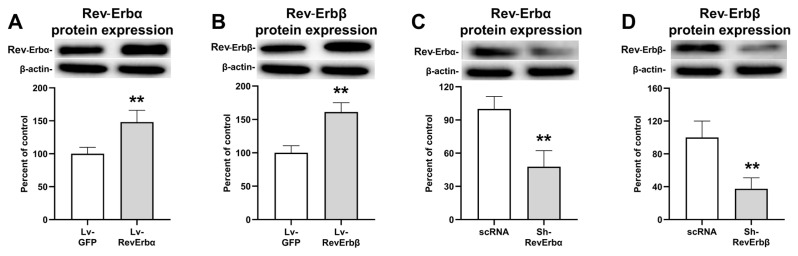
Lentiviral vectors increase or decrease of Rev-Erb proteins. Western blot analysis of overexpression of Rev-Erbα (A) and Rev-Erbβ (B) and inhibition of Rev-Erbα (C) and Rev-Erbβ (D). Representative images of western blot analysis from three independent experiments are given above their corresponding graphs. Data are represented as mean ± SD values of three independent experiments. **p < 0.01 compared with the vehicle (Lv-GFP for overexpression, scRNA for inhibition groups) treated group.

To investigate the contribution of Rev-Erbα/β to TBI outcomes, we developed specific lentiviral vectors aimed at modulating the expression of these proteins within the mouse cortex. Using western blot analysis, we quantified the expression of Rev-Erbα and β and proteins in cortical tissue samples after intracerebral injection of lentiviral vectors. This method allowed us to confirm the vectors’ capability to effectively upregulate or downregulate the target proteins, providing a foundation for their subsequent application in examining the roles of Rev-Erbα/β and in the context of TBI (Figure 1). As compared with Lv-GFP control, Lv-RevErbα and Lv-RevErbβ treatment increased the level of Rev-Erbα and Rev-Erbβ proteins significantly (p < 0.01; Figures 1A and 1B). In contrast, Sh-RevErbα and Sh-RevErbβ treatments decreased these proteins significantly compared to scRNA control (p < 0.01; Figures 1C and 1D).

### 3.2. Activation or inhibition of Rev-Erbα and β proteins plays a significant role in brain protection after TBI

**Figure 2:**
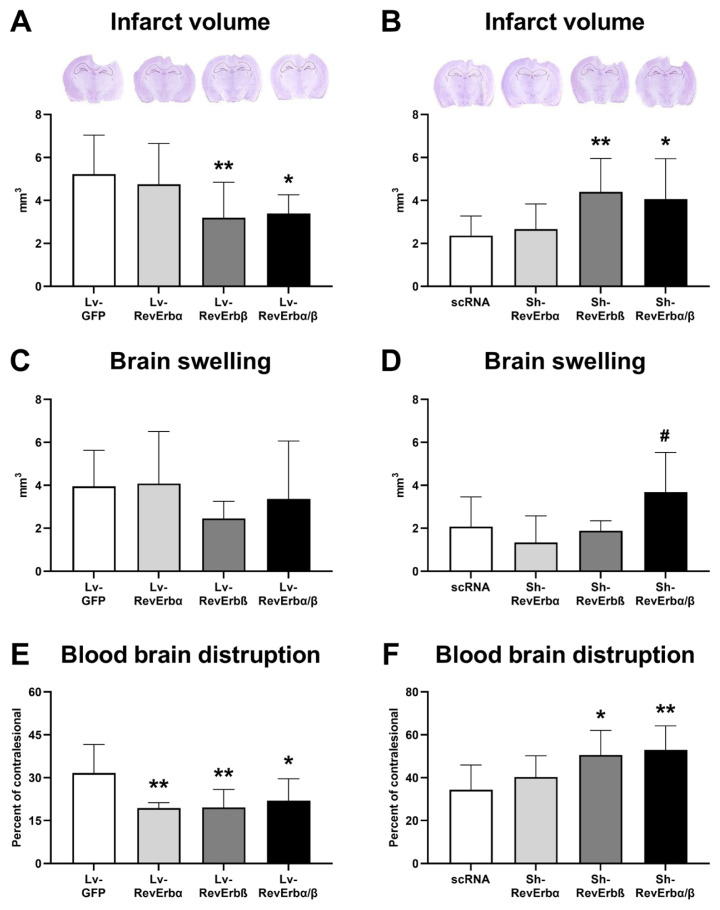
Effects of Rev-Erb proteins on brain infarct volume, swelling, and BBB permeability. Cresyl violet staining was performed to determine brain infarction (A, B) and brain swelling (C, D). To analyze BBB disruption, serum IgG extravasation was performed (E F). Data are represented as mean ± SD values. **p < 0.01/*p < 0.05 compared with the vehicle (Lv-GFP for overexpression, scRNA for inhibition groups) treated group. Scale bar, 1000 μm.

To investigate the potential protective effects of Rev-Erbα and β overexpression or inhibition on brain injury, mice were exposed to the TBI and 72 h later brain injury, swelling/edema, and BBB permeability were evaluated. Following cold-induced TBI, lesion volumes were as follows: 55.53 ± 16.74 mm^3^ for the Lv-GFP group, 52.52 ± 14.87 mm^3^ for the Lv-RevErbα group, 31.99 ± 16.44 mm^3^ for the Lv-RevErbβ group, and 33.91 ± 8.71 mm^3^ for the Lv-RevErbα/β group (Figure 2A). The Lv-RevErbβ group showed a significant reduction in lesion volume compared to the Lv-GFP group (p < 0.01). Similarly, the Lv-RevErbα/β group exhibited a significantly smaller lesion volume compared to the Lv-GFP group (p < 0.05). However, the Lv-RevErbα group did not show a statistically significant difference in lesion volume compared to the Lv-GFP group. The eta squared (η^2^) calculated for the groups was 0.347. The effect size (Cohen’s f) was 0.729, suggesting a large effect size according to Cohen’s conventions.

For the inhibition groups (scRNA, Sh-RevErbα, Sh-RevErbβ, and Sh-RevErbα/β) as shown in Figure 2B, the lesion volumes were as follows: 23.62 ± 9.17 mm^3^ for the scRNA group, 26.63 ± 11.72 mm^3^ for the Sh-RevErbα group, 49.06 ± 14.12 mm^3^ for the Sh-RevErbβ group, and 40.61 ± 18.79 mm^3^ for the Sh-RevErbα/β group. The Sh-RevErbβ group showed a significant increase in lesion volume compared to the scRNA group (p < 0.01). Similarly, the Sh-RevErbα/β group exhibited a significantly larger lesion volume compared to the scRNA group (p < 0.05). However, the Sh-RevErbα group did not show a statistically significant difference in lesion volume compared to the scRNA group. The η^2^ calculated for the groups was 0.356. The Cohen’s f was 0.744, suggesting a large effect size according to Cohen’s conventions. These results demonstrate the significant impact of Rev-Erbβ knockdown on increasing lesion volume following TBI.

The analysis of brain edema following TBI in the groups with protein overexpression (Lv-GFP, Lv-RevErbα, Lv-RevErbβ, and Lv-RevErbα/β) revealed the following mean ± standard deviation values: Lv-GFP 4.0 ± 1.7 mm^3^, Lv-RevErbα 4.1 ± 2.4 mm^3^, Lv-RevErbβ 2.5 ± 0.8 mm^3^, and Lv-RevErbα/β 3.4 ± 2.7 mm^3^ (Figure 2C). The η^2^ calculated was 0.089. The Cohen’s f was 0.313, suggesting a small to moderate effect size. These results demonstrate that the Lv-RevErbβ group had a lower brain edema volume compared to the other groups, although the differences were not statistically significant. In the groups with protein knockdown (scRNA, Sh-RevErbα, Sh-RevErbβ, and Sh-RevErbα/β), the brain edema volumes were as follows: scRNA 1.8 ± 1.7 mm^3^, Sh-RevErbα 1.3 ± 1.2 mm^3^, Sh-RevErbβ 1.9 ± 0.5 mm^3^, and Sh-RevErbα/β 3.7 ± 1.8 mm^3^ (Figure 2D). The η^2^ for this analysis was 0.297, indicating that 29.7% of the variance in brain edema was due to group differences. The Cohen’s f calculated was 0.650.

### 3.3. The role of Rev-Erbα and β in BBB permeability after TBI

The analysis of BBB permeability in the groups with protein overexpression showed the following mean ± standard deviation values: Lv-GFP 31.65 ± 9.99%, Lv-RevErbα 15.91 ± 6.17%, Lv-RevErbβ 19.58 ± 5.13%, and Lv-RevErbα/β 21.98 ± 7.63% (Figure 2E). The η^2^ for BBB permeability was 0.379, suggesting that 37.9% of the variance in permeability can be attributed to the differences between groups. The Cohen’s f was 0.781, indicating a large effect size. These results highlight significant reductions in BBB permeability in the Lv-RevErbα and Lv-RevErbβ groups compared to the Lv-GFP group, while the Lv-RevErbα/β group did not show a significant change. In the groups with protein knockdown, the BBB permeability values were as follows: scRNA 34.38 ± 11.51%, Sh-RevErbα 20.26 ± 9.98%, Sh-RevErbβ 40.26 ± 9.98%, and Sh-RevErbα/β 52.98 ± 11.89% (Figure 2F). The η^2^ for this analysis was 0.539, indicating that 53.9% of the variance in permeability was due to group differences. The Cohen’s f was 1.081, suggesting a very large effect size. These findings demonstrate a significant increase in BBB permeability in the Sh-RevErbβ and Sh-RevErbα/β groups compared to the scRNA group, with the Sh-RevErbα group showing no significant change.

### 3.4. The role of Rev-Erbα and β in the neuronal survival after TBI

**Figure 3:**
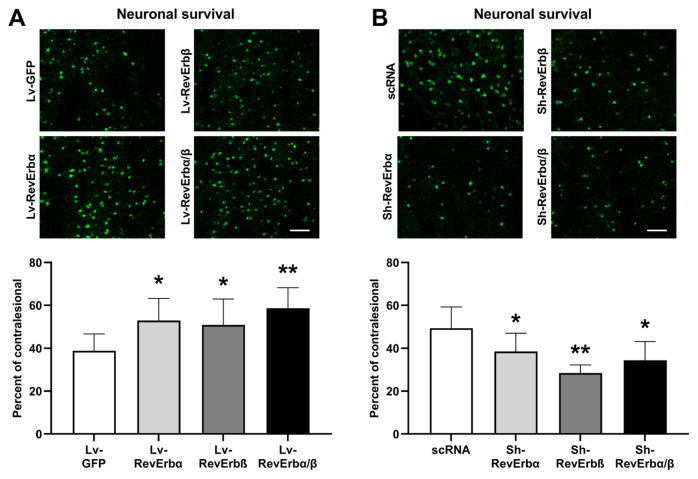
The regulation of Rev-Erb proteins plays an essential role in developing neuronal injury, which was evaluated by NeuN staining after TBI. The effect of overexpression of Rev-Erb (A) and inhibition of Rev-Erb (B) on neuronal survival was calculated as a ratio to the contralesional cortex. Data are represented as mean ± SD values. **p < 0.01/*p < 0.05 compared with the vehicle (Lv-GFP for overexpression, scRNA for inhibition groups) treated group. Scale bar, 100 μm.

To assess the impact of Rev-Erbα and β modulation on neuronal survival after TBI, brain sections were stained with NeuN. The stained sections were analyzed using a confocal microscope (LSM760, Zeiss). The results were as follows: Lv-GFP 38.83 ± 7.9%, Lv-RevErbα 52.93 ± 10.27%, Lv-RevErbβ 50.93 ± 12.02%, and Lv-RevErbα/β 58.60 ± 9.61% (Figure 3A). The η^2^ was 0.339, indicating that approximately 33.9% of the variance in neuronal survival can be attributed to the differences between groups. The Cohen’s f was 0.716, suggesting a large effect size. These results demonstrate that the Lv-RevErbα/β group had a significantly higher neuronal survival rate compared to the Lv-GFP group. For the inhibition groups, the results were as follows: scRNA 49.37 ± 9.84%, Sh-RevErbα 35.83 ± 4.48%, Sh-RevErbβ 28.60 ± 6.42%, and Sh-RevErbα/β 34.43 ± 7.84% (Figure 3B). The η^2^ for this analysis was 0.513, indicating that 51.3% of the variance in neuronal survival was due to group differences. The Cohen’s f was 1.027, suggesting a very large effect size. These findings indicate that the Sh-RevErbβ group exhibited a significantly lower neuronal survival rate compared to the scRNA group, with the Sh-RevErbα/β group also showing a significant reduction.

## 4. Discussion

The endogenous circadian clock consists of self-sustained molecular clockwork that includes the transcriptional activators Bmal1, CLOCK, and transcriptional repressors Cry1, Cry2, Per1, and Per2 [21]. Furthermore, Rev-Erb α/β inhibits the BMAL1 protein by binding to its promoter region, functioning as part of the negative feedback loop within the circadian clock machinery [11]. Although the role of Rev-Erb in the regulation of circadian rhythm is known, the number of studies showing its role in various pathophysiological processes, such as TBI, is limited. In the current study, we increased protein expression of Rev-Erbα, Rev-Erbβ, or both (Rev-Erbα/β) by lentivirus to examine the effects of Rev-Erbs on injury processes after TBI. After lentivirus injection, it takes approximately 7–10 days for the viruses to infect the brain region [25]. Therefore, in the present study, intracerebral lentivirus injections were performed 10 days before TBI. Ten days later, a cold-induced TBI model was performed, which is commonly often used to mimic brain injury seen in the clinic in experimental animals [26].

Posttraumatic brain infarction represents a critical secondary insult following TBI. Secondary brain injury processes following TBI, including neuroinflammation, oxidative stress, excitotoxicity, neurodegeneration, BBB disruption, and peripheral immune system activation, are crucial for patient prognosis and the identification of therapeutic targets. Neuroinflammation, especially prominent in the acute phase after injury, frequently leads to necrotic cell death, BBB dysfunction, and subsequent cerebral edema. Our results demonstrated that Rev-Erbβ and Rev-Erbα/β play crucial roles in the development of brain infarct volume following TBI. Notably, another important finding is that inhibition of Rev-Erbα/β significantly reduced brain swelling, highlighting its potential as a therapeutic target. It was observed that neither Rev-Erbα nor Rev-Erbβ alone was effective in mitigating brain injury outcomes; however, their combined action exhibited a synergistic effect, suggesting a complex interplay between these proteins in neuroprotection.

It is well known that TBI disrupts the integrity of the BBB, leading to increased permeability and leakage of blood-derived molecules into the brain parenchyma. This compromise of the BBB exacerbates neuroinflammation and contributes to secondary brain damage following the initial injury [29]. We performed IgG staining to determine BBB leakage. It was revealed that nuclear receptors Rev-Erbα and Rev-Erbβ have a direct effect on BBB damage after TBI. Increasing Rev-Erb proteins expression separately or together was shown to reduce BBB damage. In contrast, inhibition of the expression of these proteins increased BBB damage.

Rev-Erb has been identified as a significant modulator of neuroinflammation. Inhibiting Rev-Erb increases microglial activation and neuroinflammation, aggravating neuronal damage [30]. Conversely, activating Rev-Erbα/β with pharmacological agents has been shown to reduce LPS-induced microglial activation by blocking the NF-*κ*B pathway, thereby decreasing the production of inflammatory mediators and neuronal cytotoxicity [31]. Furthermore, Rev-Erbα/β plays a vital role in controlling peripheral inflammation, notably through its regulatory effects on NLRP3 expression and cytokine release from macrophages, critical components of TBI pathophysiology [32]. Rev-Erb’s suppression of TH17 proinflammatory responses and its influence on TLR4-mediated inflammation underscore its therapeutic potential in reducing TBI severity [33]. It is well known that circadian rhythm proteins play an essential role in disseminated neuronal injury after pathophysiological conditions such as cerebral ischemia [19–21]. In the current study, we demonstrated that Rev-Erb proteins play an essential role in the development of neuronal injury in TBI pathophysiology. Inhibition of Rev-Erb proteins increases neuronal damage, while increasing the expression of these proteins decreases neuronal damage after TBI.

Despite the promising findings of the present study, there are several limitations that need to be addressed. First, the use of a cold-induced TBI model, while common in experimental settings, may not fully replicate the complex pathophysiology of TBI seen in clinical scenarios. Second, while the overexpression and inhibition of Rev-Erb proteins were achieved using lentiviral vectors, the efficiency and specificity of these genetic modifications may vary, potentially influencing the observed outcomes. The findings of the current study suggest that targeting Rev-Erbα and Rev-Erbβ could be promising therapeutic strategies for TBI. Future research should aim to confirm these findings using additional TBI models and explore alternative methods for modulating Rev-Erb protein levels to ensure the robustness and translational potential of the results. Additionally, conducting long-term clinical trials, investigating the molecular mechanisms underlying these effects, exploring combination therapies, and developing gene therapy approaches to modulate these receptors in the brain could further validate their therapeutic potential. These efforts could pave the way for novel interventions to improve outcomes for TBI patients.

In conclusion, our findings demonstrate the critical roles of Rev-Erb proteins in modulating brain edema, BBB permeability, and neuronal survival following cold TBI. Overexpression of Rev-Erb proteins, particularly Rev-Erbβ, appears to confer protective effects, reducing brain edema, enhancing BBB integrity, and promoting neuronal survival. Conversely, the knockdown of these proteins exacerbates brain edema, disrupts BBB integrity, and decreases neuronal survival. These findings highlight the therapeutic potential of targeting Rev-Erb proteins in the treatment of TBI. Future research should focus on elucidating the molecular mechanisms underlying these effects and exploring the clinical applicability of Rev-Erb modulation in TBI therapy.

## References

[1] Dinsmore J Traumatic brain injury: an evidence-based review of management Continuing Education in Anaesthesia, Critical Care and Pain 2013 13 6 189 195 10.1093/bjaceaccp/mkt010

[2] Datta S Lin F Jones LD Pingle SC Kesari S Traumatic brain injury and immunological outcomes: the double-edged killer Future Science OA 2023 9 6 FSO864 10.2144/fsoa-2023-0037 PMC10203904 37228857

[3] Ahmed Z Chaudhary F Fraix MP Agrawal DK Epidemiology, pathophysiology, and treatment strategies of concussions: a comprehensive review Fortune Journal of Health Sciences 2024 7 2 197 215 10.26502/fjhs.178 38708028 PMC11067689

[4] LaPlaca MC Simon CM Prado GR Cullen DK CNS injury biomechanics and experimental models Progress in Brain Research 2007 161 13 26 10.1016/S0079-6123(06)61002-9 17618967

[5] Freire MAM Rocha GS Bittencourt LO Falcao D Lima RR Cellular and molecular pathophysiology of traumatic brain injury: what have we learned so far? Biology 2023 12 8 1139 10.3390/biology12081139 37627023 PMC10452099

[6] Starnes AN Jones JR Inputs and outputs of the mammalian circadian clock Biology 2023 12 4 508 523 10.3390/biology12040508 37106709 PMC10136320

[7] Finger A-M Kramer A Mammalian circadian systems: organization and modern life challenges Acta Physiologica 2021 231 3 e13548 10.1111/apha.13548 32846050

[8] Hastings MH Maywood ES Brancaccio M Generation of circadian rhythms in the suprachiasmatic nucleus Nature Reviews Neuroscience 2018 19 8 453 469 10.1038/s41583-018-0026-z 29934559

[9] Fagiani F Di Marino D Romagnoli A Travelli C Voltan D Molecular regulations of circadian rhythm and implications for physiology and diseases Signal Transduction and Targeted Therapy 2022 7 1 41 10.1038/s41392-022-00899-y 35136018 PMC8825842

[10] Patke A Young MW Axelrod S Molecular mechanisms and physiological importance of circadian rhythms Nature Reviews Molecular Cell Biology 2020 21 2 67 84 10.1038/s41580-019-0179-2 31768006

[11] Preitner N Damiola F Lopez-Molina L Zakany J Duboule D The orphan nuclear receptor REV-ERBα controls circadian transcription within the positive limb of the mammalian circadian oscillator Cell 2002 110 2 251 260 10.1016/s0092-8674(02)00825-5 12150932

[12] Abe YO Yoshitane H Kim DW Kawakami S Koebis M Rhythmic transcription of *Bmal1* stabilizes the circadian timekeeping system in mammals Nature Communications 2022 13 1 4652 10.1038/s41467-022-32326-9 PMC9399252 35999195

[13] Raza GS Sodum N Kaya Y Herzig K-H Role of circadian transcription factor Rev-Erb in metabolism and tissue fibrosis International Journal of Molecular Sciences 2022 23 21 12954 10.3390/ijms232112954 36361737 PMC9655416

[14] Amir M Chaudhari S Wang R Campbell S Mosure SA REV-ERBα regulates T_H_17 cell development and autoimmunity Cell Reports 2018 25 13 3733 3749e8 10.1016/j.celrep.2018.11.101 30590045 PMC6400287

[15] Griffett K Hayes ME Boeckman MP Burris TP The role of REV-ERB in NASH Acta Pharmacologica Sinica 2022 43 5 1133 1140 10.1038/s41401-022-00883-w 35217816 PMC9061770

[16] Wang Y Kojetin D Burris TP Anti-proliferative actions of a synthetic REV-ERBα/β agonist in breast cancer cells Biochemical Pharmacology 2015 96 4 315 322 10.1016/j.bcp.2015.06.010 26074263 PMC4526361

[17] Wu Z Liao F Luo G Qian Y He X NR1D1 deletion induces rupture-prone vulnerable plaques by regulating macrophage pyroptosis via the NF-κB/NLRP3 inflammasome pathway Oxidative Medicine and Cellular Longevity 2021 2021 1 15 10.1155/2021/5217572 PMC8702349 34956438

[18] Lee S-B Yang HO Sinapic acid ameliorates REV-ERB α modulated mitochondrial fission against MPTP-induced Parkinson’s disease model Biomolecules & Therapeutics 2022 30 5 409 417 10.4062/biomolther.2022.020 35611585 PMC9424337

[19] Beker MÇ Kılıç E The role of circadian rhythm in the regulation of cellular protein profiles in the brain Turkish Journal of Medical Sciences 2021 51 5 2705 2715 10.3906/sag-2010-336 33356029

[20] Beker MC Caglayan B Yalcin E Caglayan AB Turkseven S Time-of-day dependent neuronal injury after ischemic stroke: implication of circadian clock transcriptional factor Bmal1 and survival kinase AKT Molecular Neurobiology 2018 55 3 2565 2576 10.1007/s12035-017-0524-4 28421530

[21] Beker MC Caglayan B Caglayan AB Kelestemur T Yalcin E Interaction of melatonin and Bmal1 in the regulation of PI3K/AKT pathway components and cellular survival Scientific Reports 2019 9 1 19082 10.1038/s41598-019-55663-0 31836786 PMC6910929

[22] Verma AK Singh S Rizvi SI Aging, circadian disruption and neurodegeneration: interesting interplay Experimental Gerontology 2023 172 112076 10.1016/j.exger.2022.112076 36574855

[23] Fifel K Videnovic A Circadian and sleep dysfunctions in neurodegenerative disorders—an update Frontiers in Neuroscience 2020 14 627330 10.3389/fnins.2020.627330 33536872 PMC7848154

[24] Nassan M Videnovic A Circadian rhythms in neurodegenerative disorders Nature Reviews Neurology 2022 18 1 7 24 10.1038/s41582-021-00577-7 34759373

[25] Beker M Caglayan AB Beker MC Altunay S Karacay R Lentivirally administered glial cell line-derived neurotrophic factor promotes post-ischemic neurological recovery, brain remodeling and contralesional pyramidal tract plasticity by regulating axonal growth inhibitors and guidance proteins Experimental Neurology 2020 331 113364 10.1016/j.expneurol.2020.113364 32454038

[26] Ciftci E Karacay R Caglayan A Altunay S Ates N Neuroprotective effect of lithium in cold-induced traumatic brain injury in mice Behavioural Brain Research 2020 392 112719 10.1016/j.bbr.2020.112719 32479849

[27] Beker MC Caglayan AB Altunay S Ozbay E Ates N Phosphodiesterase 10A is a critical target for neuroprotection in a mouse model of ischemic stroke Molecular Neurobiology 2022 59 1 574 589 10.1007/s12035-021-02621-5 34735672

[28] Caglayan B Kilic E Dalay A Altunay S Tuzcu M Allyl isothiocyanate attenuates oxidative stress and inflammation by modulating Nrf2/HO-1 and NF-κB pathways in traumatic brain injury in mice Molecular Biology Reports 2019 46 241 250 10.1007/s11033-018-4465-4 30406889

[29] Wu Y Wu H Guo X Pluimer B Zhao Z Blood-brain barrier dysfunction in mild traumatic brain injury: evidence from preclinical murine models Frontiers in Physiology 2020 11 1030 10.3389/fphys.2020.01030 32973558 PMC7472692

[30] Griffin P Dimitry JM Sheehan PW Lananna BV Guo C Circadian clock protein Rev-erbα regulates neuroinflammation Proceedings of the National Academy of Sciences 2019 116 11 5102 5107 10.1073/pnas.1812405116 PMC6421453 30792350

[31] Guo D-k Zhu Y Sun H-y Xu X-y Zhang S Pharmacological activation of REV-ERBα represses LPS-induced microglial activation through the NF-κB pathway Acta Pharmacologica Sinica 2019 40 1 26 34 10.1038/s41401-018-0064-0 29950615 PMC6318300

[32] Pourcet B Zecchin M Ferri L Beauchamp J Sitaula S Nuclear receptor subfamily 1 group D member 1 regulates circadian activity of NLRP3 inflammasome to reduce the severity of fulminant hepatitis in mice Gastroenterology 2018 154 5 1449 1464e20 10.1053/j.gastro.2017.12.019 29277561 PMC5892845

[33] Chang C Loo C-S Zhao X Solt LA Liang Y The nuclear receptor REV-ERBα modulates Th17 cell-mediated autoimmune disease Proceedings of the National Academy of Sciences 2019 116 37 18528 18536 10.1073/pnas.1907563116 PMC6744854 31455731

